# Analysis of gut microbiome profiles in common marmosets (*Callithrix jacchus*) in health and intestinal disease

**DOI:** 10.1038/s41598-022-08255-4

**Published:** 2022-03-15

**Authors:** Alexander Sheh, Stephen C. Artim, Monika A. Burns, Jose Arturo Molina-Mora, Mary Anne Lee, JoAnn Dzink-Fox, Sureshkumar Muthupalani, James G. Fox

**Affiliations:** 1 Division of Comparative Medicine, Massachusetts Institute of Technology, Cambridge, MA USA; 2 Centro de Investigación en Enfermedades Tropicales (CIET), Universidad de Costa Rica, San José, Costa Rica; 3 Department of Biological Sciences, Wellesley College, Wellesley, MA USA; 4 Present Address: Merck Research Laboratories, Merck, South San Francisco, CA USA

**Keywords:** Gastroenterology, Gastrointestinal diseases, Gastrointestinal models, Bacteria, Microbial communities, Machine learning

## Abstract

Chronic gastrointestinal (GI) diseases are the most common diseases in captive common marmosets. To understand the role of the microbiome in GI diseases, we characterized the gut microbiome of 91 healthy marmosets (303 samples) and 59 marmosets diagnosed with inflammatory bowel disease (IBD) (200 samples). Healthy marmosets exhibited “humanized,” *Bacteroidetes*-dominant microbiomes. After up to 2 years of standardized diet, housing and husbandry, marmoset microbiomes could be classified into four distinct marmoset sources based on *Prevotella* and *Bacteroides* levels. Using a random forest (RF) model, marmosets were classified by source with an accuracy of 93% with 100% sensitivity and 95% specificity using abundance data from 4 *Prevotellaceae* amplicon sequence variants (ASVs), as well as single ASVs from *Coprobacter, Parabacteroides, Paraprevotella, Phascolarctobacterium, Oribacterium* and *Fusobacterium.* A single dysbiotic IBD state was not found across all marmoset sources, but IBD was associated with lower alpha diversity and a lower *Bacteroides*:*Prevotella copri* ratio within each source. IBD was highest in a *Prevotella*-dominant cohort, and consistent with *Prevotella*-linked diseases, pro-inflammatory genes in the jejunum were upregulated. RF analysis of serum biomarkers identified serum calcium, hemoglobin and red blood cell (RBC) counts as potential biomarkers for marmoset IBD. This study characterizes the microbiome of healthy captive common marmosets and demonstrates that source-specific microbiomes can be retained despite standardized diets and husbandry practices. Marmosets with IBD had decreased alpha diversity and a shift in the ratio of *Bacteroides:Prevotella copri* compared to healthy marmosets*.*

## Introduction

*Callithrix jacchus* is a diurnal, arboreal, New World, non-human primate (NHP). Adults can weigh 300–500 g with average lifespans of 5 to 12 years of age in research colonies^1–4^. Due to their size, high fecundity and similarity to humans, they have become animal models for aging, vision, behavioral neuroscience, multiple sclerosis, auditory research, neurodegenerative diseases, and toxicology^5^. Gastrointestinal (GI) diseases are the most common and widespread clinical finding in captive common marmosets^6,7^. IBD prevalence is reported to be as high as 28–60% in captive marmosets and presents with diarrhea, weight loss, enteritis, muscle atrophy, alopecia, hypoproteinemia, anemia, elevated liver enzymes, failure to thrive and mortality^6,8^. Histologic analysis can further define the IBD diagnosis into chronic lymphocytic enteritis (CLE) based on findings, such as small intestinal localization, shortened villi, crypt epithelial hyperplasia, and lymphocytic infiltration of the lamina propria^6,7^. Previously reported marmoset biomarkers of IBD include calprotectin and matrix metalloproteinase 9^9,10^; however, clinical interventions involving glucocorticoids, gluten-free diets, Giardia treatment, etc. have yielded mixed results^11–13^.

In healthy individuals, the human GI tract harbors trillions of microorganisms from at least 400 species that compose the intestinal microbiota, which influences many physiological functions such as extracting nutrients, maintaining the gut mucosal barrier, training immune cells and protecting against pathogens^14–16^. Studies in humans have shown that a disturbance in the gut microbiota, known as dysbiosis, is associated with a wide spectrum of diseases including IBD, irritable bowel syndrome, obesity, psoriasis, rheumatoid arthritis, autism spectrum disorders, and *Clostridioles difficile* infection^16–18^. Over 3.5 million people worldwide are affected by IBD, a chronic gastrointestinal (GI) inflammatory disease triggered by interactions between host, microbes and the environment^19–23^. Two common forms of IBD are Crohn’s disease (CD), which can affect the small and large intestines, and ulcerative colitis (UC), which localizes to the large intestine. Over 200 genomic loci may confer increased IBD risk in humans, with many of these genes associated with regulating host-microbe interactions^19^. Changes in the intestinal microbiota observed in IBD patients have included reduction of short chain fatty acid (SCFA) producing bacteria, reduced alpha diversity, decreased *Firmicutes* abundance, and increased abundance of facultative anaerobes, *Proteobacteria* and *Bacteroidetes*^20–22,24–26^.

Understanding differences in the gut microbiome between health and disease states could eventually lead to insight on the etiology and pathogenesis of the disease, novel biomarkers or potentially lead to therapeutics for the disease^27^. Currently, few peer reviewed reports on the marmoset microbiome are available but they lack the cohort size evaluated in our current study^28^. These studies have not evaluated the effect of IBD on the microbiome of a large cohort of marmosets^29–34^. A study by Shigeno et al. compared bacteria in healthy marmosets and marmosets with chronic diarrhea, but used low resolution terminal restriction fragment-length polymorphism (T-RFLP) to compare both groups^35^. To our knowledge, there is a single study longitudinally tracking the microbiome of captive common marmosets over multiple months, but their focus was the evolution of the microbiome in marmoset breeding pairs^34^. These studies have demonstrated that captivity and diets fed to captive marmosets have been associated with microbial diversity loss, shifts in the Firmicutes:Bacteroidetes ratio, and increased GI disease and mortality^36–38^. Dietary specialists, such as marmosets, are more susceptible to captivity-associated dietary changes^37^. Marmosets are exudivores that consume large amounts of indigestible oligosaccharides from tree gums^39^ and may harbor specific gut microbes dedicated to carbohydrate metabolism.

The goal of this study was to characterize the microbiome of healthy captive marmosets and evaluate differences in the microbiome and blood samples between healthy marmosets and marmosets with IBD to identify potential biomarkers of marmoset IBD. To this end, this study evaluated longitudinal microbiome, serum chemistry and complete blood count (CBC) samples from a large cohort of healthy marmosets (n = 91) and marmosets with IBD (n = 59) with a sex ratio of 0.49, collected during physical examinations or necropsies over a 2-year period. Additionally, our study tracked the original source of each imported marmosets allowing us to evaluate the effect of diet, husbandry and source on the healthy microbiome. ‘Healthy’ controls were defined as individuals not clinically diagnosed with IBD and not receiving chronic drug treatments during the study period. Based on this analysis, unique microbial profiles were associated with the four sources that originally populated the MIT marmoset colony. We also identified shifts in the *Bacteroides:Prevotella copri* ratio and decreases in serum calcium, hemoglobin, and red blood cell counts that associated with IBD in marmosets. These may serve as marmoset biomarkers for IBD, and reflect the potential of marmosets as an animal model of Crohn’s disease.

## Results

### Microbial diversity in the intestinal microbiota of the common marmoset

**Figure 1:**
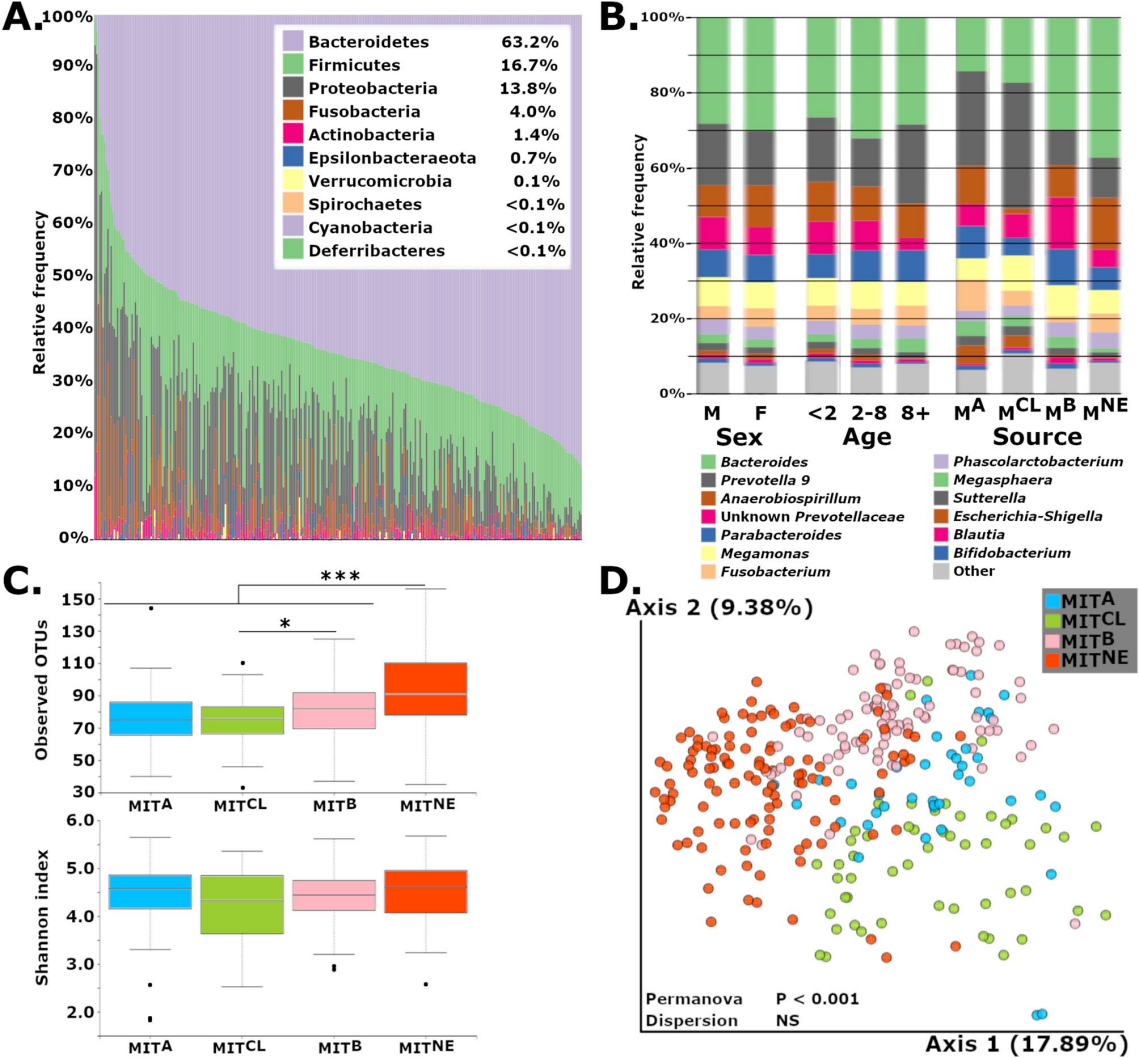
(**A**) Gut microbiome profiles of healthy, common marmosets at the phylum level exhibit a Bacteroidetes-dominant and human-like microbiome. (**B**) Averaged relative abundances at the genus level show differences associated with source but few differences based on sex or age. (**C**) Observed OTUs were increased in MIT^NE^ vs. all sources, and MIT^B^ vs. MIT^A^ and MIT^CL^, but metrics involving evenness, such as Shannon’s diversity index, showed no difference. Boxplots encompass the 25th and 75th percentiles of the distribution with the horizontal bar representing the median. *P < 0.05; **P < 0.01 and ***P < 0.001. (**D**) PCoA plot using Unweighted UniFrac metric shows clustering of microbiome profiles based on marmoset source.

303 samples from 91 healthy marmosets were analyzed to determine the normal microbiota of captive common marmosets within the MIT colony (Table 1). 99% of the average microbial abundance in feces was captured by Bacteroidetes, Firmicutes, Proteobacteria, Fusobacteria and Actinobacteria (Fig. 1A). The microbiome profile observed in healthy, MIT marmosets resembles the microbiome observed in human stool with dominance of the phylum Bacteroidetes (average 63.2%), followed distantly by Firmicutes and Proteobacteria^15^. As observed in humans^15^, Bacteroidetes abundance varied significantly, ranging from 8 to 86%. Bacteroidetes were predominantly represented by *Bacteroides*, *Prevotella 9* and *Parabacteroides*. The most abundant Firmicutes were *Megamonas, Megasphaera,* and *Phascolartcobacterium*. *Anaerobiospirillum, Sutterella* and *Escherichia-Shigella* were the most common Proteobacteria. Notably, *Bifidobacterium* was present in low abundance compared to other reported marmoset microbiomes^29,30^ (Supp. Table 1).

**Table 1:** Description of microbiome sample demographics.

		Unique healthy animals	Healthy samples	Unique IBD animals	IBD samples
Sex	Male	46	156	30	95
	Female	45	147	29	105
Age^a^	2 and under	NA	145	NA	36
	2 to 8	NA	139	NA	131
	Over 8	NA	19	NA	33
Source	MIT^NE^	37	117	24	89
	MIT^B^	27	94	8	30
	MIT^CL^	19	53	24	61
	MIT^A^	8	39	3	20
Type^b^	Rectal	90	207	48	111
	Fecal	56	96	50	89

^a^Number of animals not reported as samples were collected over 2 year period and animals spanned multiple age groups.

^b^Fecal and Rectal Swabs were often collected from the same animal, so number of animals will be higher.

### Source population impacted microbiome diversity

Having established the baseline microbiome for healthy, MIT marmosets, we explored the effects of age, sex, and original source, and found that source strongly influenced microbial composition (Fig. 1B). MIT’s colony originally imported marmosets from four sources designated MIT^A^, MIT^B^, MIT^CL^ and MIT^NE^. Marmosets were housed in two buildings and provided standardized diet, husbandry and veterinary care, but were only co-housed with same-source animals for the duration of the study. Using multiple estimators for alpha diversity, we noted that species richness estimators significantly differed between healthy marmosets by source, but not sex or age. MIT^NE^ marmosets had higher observed OTUs and Chao1 values compared to other sources (P < 0.001 vs. each source, both metrics) (Fig. 1C, Supp. Fig. 1). MIT^B^ had significantly higher alpha diversity compared to MIT^CL^ (observed OTUs, P < 0.05; Chao1, P < 0.01) and MIT^A^ (Chao1, P < 0.05). However, differences were not observed when accounting for evenness (Shannon diversity or Pielou’s evenness). Clustering of samples based on source (Unweighted UniFrac: PERMANOVA, P < 0.001; beta-dispersion, NS) (Fig. 1D), but not sex or age, was also observed (Supp. Fig. 2).

We next identified 63 differentially abundant genera between the 4 sources in the lower gut using ANCOM (Analysis of Composition of Microbiomes). 13 genera were present at relative abundances greater than 1% in at least one source (Supp. Table 2). High abundance of *Bacteroides* characterized MIT^NE^ and MIT^B^ samples, while MIT^CL^ and MIT^A^ were primarily colonized by genus *Prevotella 9* (Fig. 1B). The *Bacteroidaceae*:*Prevotellaceae* ratios for MIT^A^, MIT^CL^, MIT^B^, and MIT^NE^ (0.44, 0.39, 1.23 and 2.17, respectively) emphasize source-associated differences reflected in these two genera. *Anaerobiospirillum*, another highly abundant genus, represented 8.5–13.8% of bacterial in three sources but had low numbers in MIT^CL^ marmosets (1.5%).

### Identification of microbial biomarkers defining source-associated microbiomes using machine learning

**Figure 2:**
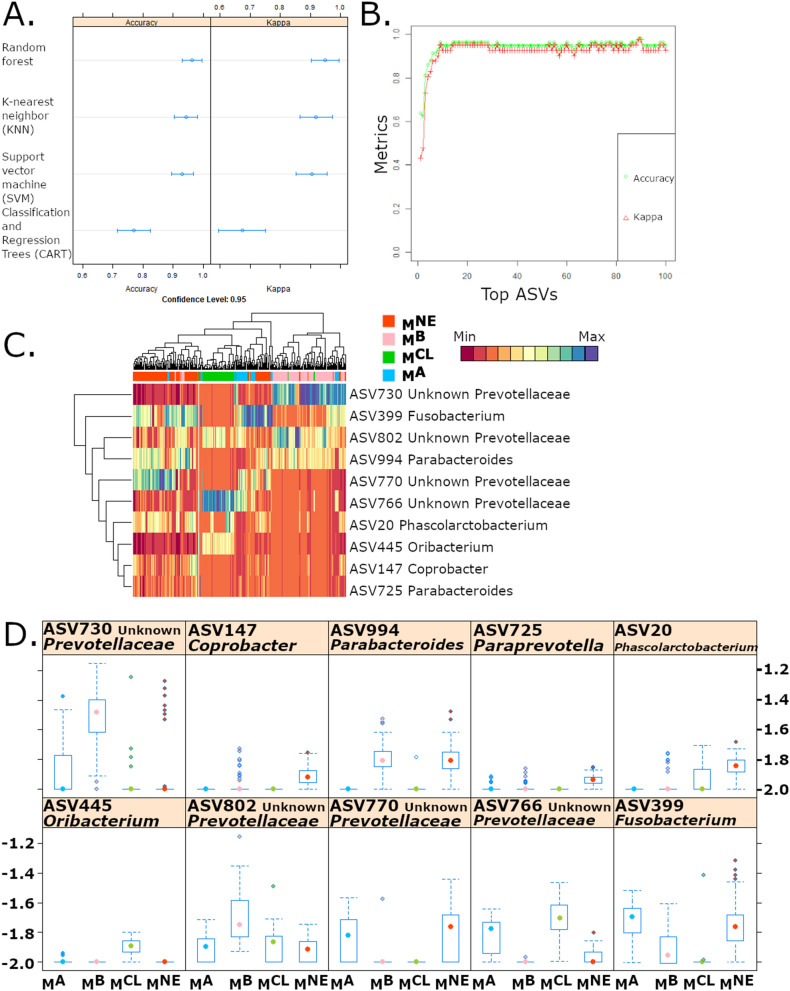
(**A**) Comparison of classifier models used to classify healthy microbiomes based on source included random forest (RF), K-nearest neighbor (KNN), support vector machines (SVM), and classification and regression trees (CART). RF consistently outperformed the other classifiers. (**B**) Accuracy and Kappa of RF model stabilizes with 10 variables. (**C**) Heatmap of ASV abundances showing classification of data using 10 ASVs. Color bar on top indicates source. (**D**) Boxplots of 10 ASVs selected by RF model show source-specific differences.

As data indicated that distinct microbiome profiles could be conserved when same-source marmosets were co-housed, the microbiome was further analyzed using four machine learning algorithms (random forest (RF), support vector machine (SVM), classification and regression trees (CART) and k-nearest neighbor (KNN)) to determine the Amplicon Sequence Variants (ASV) that best identified each of the four sources. Models were evaluated based on the accuracy and kappa of each algorithm (Fig. 2A). Accuracy measures the percentage of correctly classified instances by comparing the testing set’s ground truth data with the model’s predictions, while kappa compares sample classification agreement and accounts for the hypothetical probability of random agreements. Kappa values greater than 0.40 reflect moderate or substantial agreement between the model and ground truth. Comparison of the four classification models shows that RF analysis provided the highest accuracy and kappa values when classifying microbiome profiles based on the source. Focusing on the RF model, we then evaluated the stability of three metrics (accuracy, kappa and F1 scores) to determine the least number of ASVs that maximized the three metrics. F1 score evaluates the model’s utility using both precision and recall (or sensitivity). We selected a 10 ASV model that presented the highest levels of accuracy, F1 and kappa observed with the RF model (Fig. 2B). Source-specific differences used by the classification model to distinguish the sources were observed when representing the data using a heatmap and boxplots (Fig. 2C,D). Of the 10 ASVs, 4 ASVs were in the family *Prevotellaceae*, as well as a single ASV from *Coprobacter, Parabacteroides, Paraprevotella, Phascolarctobacterium, Oribacterium* and *Fusobacterium*. The optimized model achieved an accuracy of 93% with 100% sensitivity and 95% specificity. The RF model confirmed that despite importation and assimilation, unique source-specific signature microbiota were retained by cohousing same-source animals.

### Effects of IBD on the microbiome

**Figure 3:**
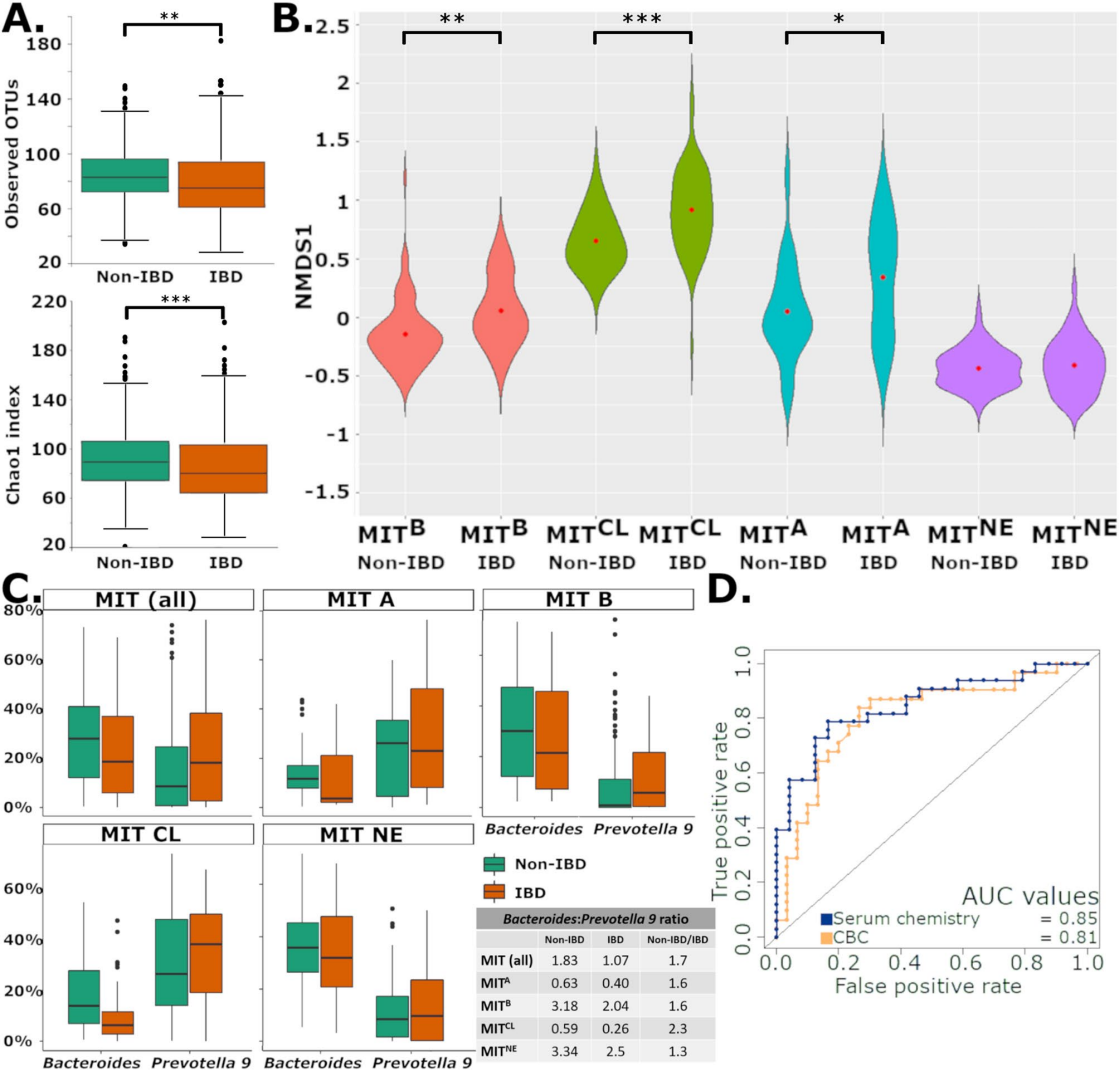
(**A**) Decreased richness was observed in IBD marmosets (Observed OTUs and Chao1) compared to non-IBD marmosets similar to what is observed in humans. (**B**) Increases in PC1 relative to source-specific, non-IBD controls were observed in 3 of 4 sources. Red dot in violin plots represents the mean. (**C**) *Bacteroides* and *Prevotella 9* levels are shown by source and IBD status. A lower overall and source-specific *Bacteroides:Prevotella 9* ratio is observed in IBD cases regardless of source-specific differences in abundances of these two genera. (**D**) AUC of ROC for random forest models using serum chemistry and CBC show strong performance of models in classifying IBD progressors and non-progressors. Boxplots encompass the 25th and 75th percentiles of the distribution with the horizontal bar representing the median. *P < 0.05; **P < 0.01 and ***P < 0.001.

Despite the observed differences in microbiome composition between the four sources, IBD was diagnosed in marmosets from all sources at MIT with varied prevalence (MIT^CL^, 55%; MIT^NE^, 29%; MIT^A^, 27%; and MIT^B^, 22%) (Supp. Table 3). To study the effects of marmoset IBD on the microbiome, we focused on marmosets categorized as “non-progressors” (n = 91) or “progressors” (n = 59). “Progressors” were diagnosed with or developed IBD during the study, while “non-progressors” were healthy or were diagnosed with non-IBD diseases after the study. IBD diagnosis was determined by clinicians, veterinarians, and pathologists using each marmoset’s medical history (episodes of chronic of diarrhea, weight loss, chronic use of budesonide, low albumin levels, and other factors indicative of IBD) and histological analysis of tissues post-mortem (when available) to confirm the diagnosis of IBD. Once a diagnosis of IBD was determined through either the clinical or pathological assessment, all samples for that animal were classified as “progressors” for this study, as the exact date of IBD onset was not possible to determine. Across the colony, alpha diversity metrics focusing on microbiome richness were lower in IBD progressors (Chao1, P < 0.001; Observed OTUs, P < 0.001) (Fig. 3A), but when accounting for microbiome richness and evenness, we did not observe changes in alpha diversity metrics (Shannon’s index and Pielou’s evenness). We used PCA to determine if progressors converged at a common dysbiotic state, but similar to human IBD studies^21,23,40^, no single microbial community structure was consistently associated with IBD across all sources (Supp. Fig. 3a). Despite clinical IBD, the community structures observed in the microbiome remained dependent on source. However, positive shifts along the first principal component (PC) were observed locally within individual sources (Fig. 3B, Supp. Fig. 3a). Statistical analysis of the differences in PC1 within each individual source exhibited significant differences in PC1 values between healthy and IBD cases from the same source in 3 of 4 sources (MIT^B^, P < 0.01; MIT^CL^, P < 0.001; MIT^A^, P < 0.05; MIT^NE^, P = 0.6) (Fig. 3B).

While no shared dysbiotic IBD state existed, we hypothesized that source-specific, healthy states could become source-specific, IBD states through similar perturbations of the microbiome. To identify IBD-associated changes in the microbiome within source-specific subsets, ASVs correlated with PC1 were examined. Five *Prevotellaceae* ASVs (*Prevotella 9* and unclassified genera) and 3 *Megasphaera* ASVs were positively correlated with PC1, while 5 *Parabacteroides* ASVs and 3 *Bacteroides* ASVs were anti-correlated to PC1. We also utilized the framework developed to create the RF model that classified healthy marmosets based on source (Fig. 2) to develop 4 new models to classify progressors and non-progressors using data from (a) the entire colony, (b) MIT^B^, (c) MIT^CL^ and (d) MIT^NE^ (MIT^A^ was excluded due to insufficient n). As we did previously, we ranked ASVs for each of the 4 models based on their ability to classify progressors and non-progressors. To find ASVs that were shared amongst the 4 RF models, we compared the overlap of the top 25 ASVs from each model. We identified 8 ASVs that were shared by 3 or more models (Supp. Table 4). The shared ASVs belonged to *Sutterella* (3), *Megamonas* (2)*, Bacteroides, Asteroleplasma,* and *Prevotella 9*, and suggest that shifts in these ASVs are informative about IBD status in the marmoset microbiome. As both analyses highlighted the importance of *Bacteroides* and *Prevotella 9*, two important genera in the human gut microbiome^15,41^, we examined the relationship between *Bacteroides* and *Prevotella 9* in marmoset IBD. Using BLAST, 99.93% of *Prevotella 9* reads matched *P. copri* with a > 99% identity. In contrast, *Bacteroides* reads matched multiple species including *B. plebeius* (48.3%), *B. vulgatus* (16.8%), *B. uniformis* (6.4%), *B. dorei* (4.3%), *B. massiliensis* (3,2%), *B. thetaiotaomicron* (1.9%), *B. ovatus* (1.9%) and *B. coprocola* (1%). Comparing the relative abundance of these genera between progressors and non-progressors, we observed decreases in *Bacteroides*, while *Prevotella 9* remained level or increased (Fig. 3C). As *Bacteroides* and *Prevotella* compete for the same niche in the gut, we evaluated the ratio of average *Bacteroides* abundance to average *Prevotella 9* abundance. For the entire colony, this ratio was 1.83 in non-progressors and 1.07 in IBD progressors, yielding a non-progressor/progressor ratio of 1.7. A similar ratio is observed when categorizing marmosets by source, with a larger ratio observed in non-progressors relative to progressors. These results imply that marmosets with IBD may experience a relative decrease in *Bacteroides* spp. and conversely a relative increase in *P. copri* relative abundance (Fig. 3C).

### Effects of IBD on blood analysis

To identify other potential biomarkers, serum chemistry and CBC data collected in the course of clinical examinations from our previous publication^42^ and medical records to develop RF models using either serum chemistry or CBC data from IBD progressors and non-progressors to identify other potential biomarkers. Unlike the microbiome data, source-dependent clustering was not observed in marmoset serum chemistry or CBC PCA plots (Supp. Fig. 3b,c). As source had less impact on serum chemistry and CBC data, these RF classifiers were trained solely on IBD status. The serum chemistry RF model was optimized with 7 parameters (calcium, GGT, albumin, A:G ratio, amylase, cholesterol, and alkaline phosphatase), and had an accuracy of 77%, a sensitivity of 79%, a specificity of 76% and AUC of 0.85 (Fig. 3D). The optimized CBC RF model used hemoglobin (HGB), red blood cell (RBC) count, red blood cell distribution width (RDW), mean platelet volume (MPV) and neutrophil %, and had an accuracy of 77%, a sensitivity of 73%, a specificity of 83% and AUC of 0.81 (Fig. 3D). Based on the importance assigned to each variable by the models, the most informative variables for the classification of marmoset IBD were calcium, hemoglobin, and RBC, which showed decreased levels in marmosets with IBD compared to the healthy cohort (Supp. Fig. 4).

### Effects of GI disease on gene expression of the small intestine

**Figure 4:**
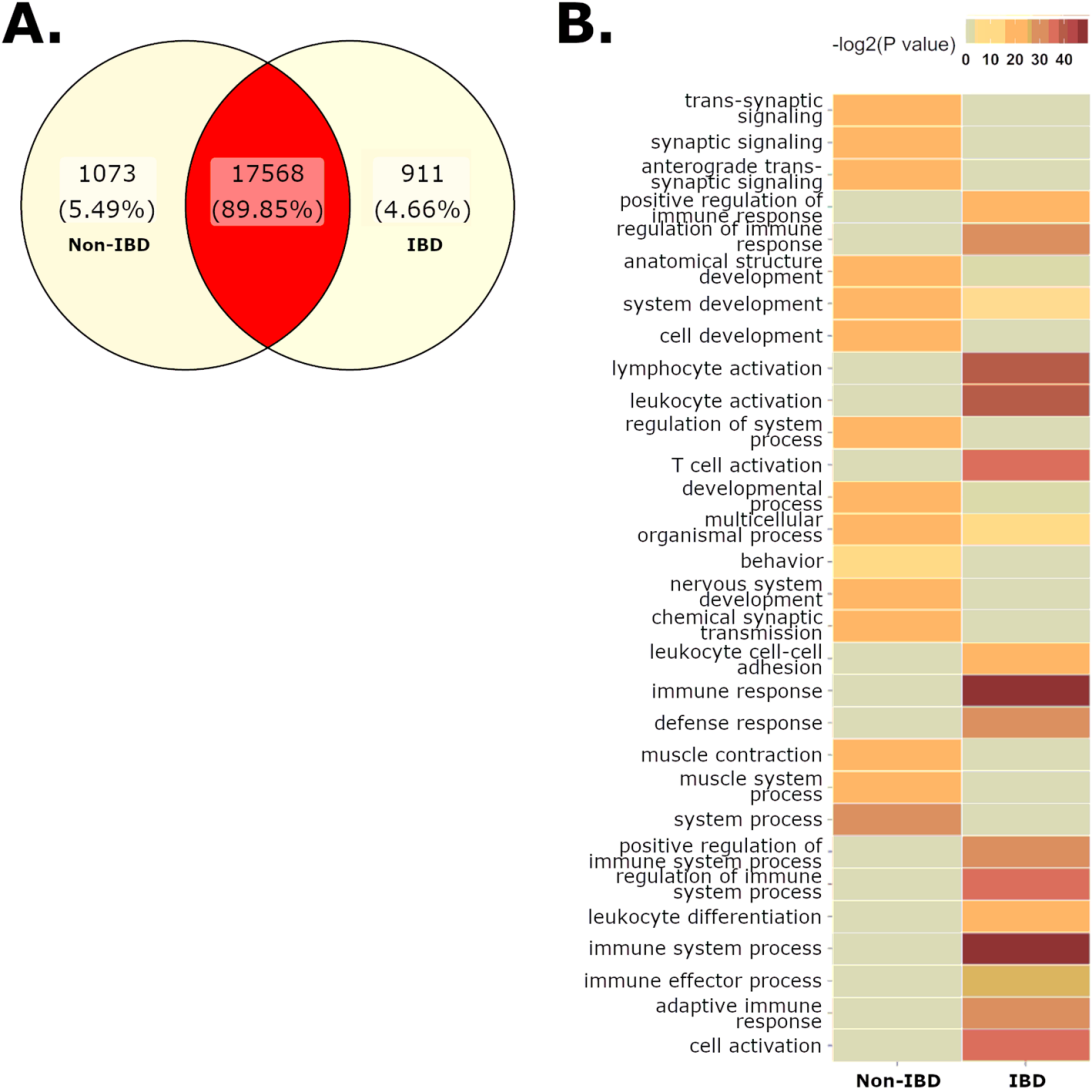
(**A**) Differentially expressed genes (DEG) (FDR < 0.05) in the jejunum of non-IBD and IBD cases. (**B**) IBD samples are enriched in GO sets associated with immunity and immune cell activation.

We then tested whether IBD significantly altered marmoset transcriptomic profiles using RNA sequencing (RNAseq) on jejunum samples from IBD (n = 3) or non-IBD (n = 3) marmosets. The jejunum was selected to evaluate the effects of IBD, as it is strongly affected during IBD^6^. While the non-IBD marmosets were not clinically healthy, the jejunum of these marmosets presented minimal pathology^43^, and was determined suitable to be used as “non-IBD,” jejunum controls. 1984 differentially expressed genes (DEGs) were identified when comparing jejunums from IBD and non-IBD marmosets (Fig. 4A, Supp. Table 5) following the exclusion of an outlier IBD sample that did not cluster with other samples (Supp. Fig. 5). GO annotations were assigned to 1586 DEGs, and the top 15 BP are summarized in Table 2a (complete list—Supp. Table 6). The jejunum of IBD animals enriched GOs associated with host immunity, such as T cell activation, adaptive immune responses, and regulation of immune response (Fig. 4B, Supp. Fig. 5). Genes associated with killer cell lectin-like receptors (*KLRB1, KLRC1, KLRC2, KLRF1,* and *KLRK1*) and antimicrobial responses (*LCN2*, *LYZ*, and *MUC20*) were upregulated in the jejunum of IBD marmosets. Genes involved in the adaptive immunity and T cell activation (*EOMES, PRF1, IFNG, FYN, CD160, CD244, CD3G, TBX21, CD27, PTPRC,* and *IL18R1*) had increased expression in IBD samples (Supp. Table 5)*.* In non-IBD animals, top GOs associated with homeostatic functions, such as synaptic signaling, development, and muscle contraction (Table 2b, Supp. Fig. 6).

**Table 2:** Top gene ontology sets in IBD.

GO ID	Term	Ont	N	Up	Down	P.Up	P.Down
**(a) Biological processes upregulated in the Jejunum in IBD**							
GO:0002376	Immune system process	BP	2197	90	286	1	7.30E−58
GO:0006955	Immune response	BP	1473	39	227	1	7.75E−57
GO:0045321	Leukocyte activation	BP	949	24	165	1	7.62E−47
GO:0046649	Lymphocyte activation	BP	516	11	119	0.9999998	4.13E−46
GO:0042110	T cell activation	BP	361	7	97	0.9999956	1.32E−43
GO:0002682	Regulation of immune system process	BP	1154	40	177	0.9999999	1.42E−42
GO:0001775	Cell activation	BP	1071	37	169	0.9999997	3.75E−42
GO:0002250	Adaptive immune response	BP	282	3	81	0.9999995	9.97E−39
GO:0002684	Positive regulation of immune system process	BP	816	20	136	1	5.02E−36
GO:0050776	Regulation of immune response	BP	758	17	130	1	1.07E−35
GO:0006952	Defense response	BP	1139	48	161	0.9999512	6.45E−34
GO:0002252	Immune effector process	BP	890	19	135	1	2.64E−31
GO:0050778	Positive regulation of immune response	BP	601	10	104	1	1.02E−28
GO:0002521	Leukocyte differentiation	BP	402	9	83	0.9999927	2.24E−28
GO:0007159	Leukocyte cell–cell adhesion	BP	254	6	64	0.9996237	4.13E−27
**(b) Biological processes upregulated in the Jejunum in non-IBD**							
GO:0003008	System process	BP	1229	204	64	3.89E−36	5.12E−01
GO:0099537	Trans-synaptic signaling	BP	538	105	20	8.64E−24	9.59E−01
GO:0032501	Multicellular organismal process	BP	5121	490	327	1.40E−23	7.31E−07
GO:0099536	Synaptic signaling	BP	543	105	20	1.85E−23	9.63E−01
GO:0048731	System development	BP	3556	373	229	4.31E−23	7.45E−05
GO:0044057	Regulation of system process	BP	444	92	17	8.25E−23	9.29E−01
GO:0098916	Anterograde trans-synaptic signaling	BP	530	102	20	1.23E−22	9.51E−01
GO:0007268	Chemical synaptic transmission	BP	530	102	20	1.23E−22	9.51E−01
GO:0006936	Muscle contraction	BP	267	66	10	7.02E−21	8.93E−01
GO:0032502	Developmental process	BP	4618	444	281	1.20E−20	4.77E−04
GO:0007399	Nervous system development	BP	1847	226	95	1.33E−20	5.61E−01
GO:0048468	Cell development	BP	1674	210	98	2.52E−20	1.09E−01
GO:0048856	Anatomical structure development	BP	4314	418	263	1.43E−19	7.56E−04
GO:0003012	Muscle system process	BP	340	73	11	3.50E−19	9.69E−01
GO:0007275	Multicellular organism development	BP	3945	389	245	4.19E−19	4.20E−04

## Discussion

GI diseases are the most prevalent clinical disease in captive common marmosets^6,7,44^. Recent literature demonstrates that housing in captive environments affects NHP microbiome composition, reduces alpha diversity, and alters host responses to disease^36,37,45^. In captivity, NHP microbiomes lose distinctive, wild microbiota and become dominated by *Prevotella* and *Bacteroides*, the most abundant genera in the modern human gut microbiome^15,36,41^. In the largest marmoset microbiome study to date, we examined the microbiome of both healthy marmosets and marmosets presenting clinically with IBD. The microbiome of healthy captive marmosets resembles the human microbiome, as *Bacteroides* and *Prevotella 9* were the most abundant genera with levels similar to those observed in human feces^15,41^. In humans, *Prevotella* and *Bacteroides* abundances are anticorrelated, signifying that competitive advantages in metabolism determine the dominant bacteria^46,47^. *Prevotella* increases have been associated with high-fiber, plant-based diets and non-industrialized populations, while *Bacteroides* increases were linked to Westernized populations with diets rich in animal fat and protein^46,47^. Diets influence levels of fibers, fermentation products, SCFA and bile acids (BA), which determine bacterial communities^47^. As our marmosets were fed a standardized diet, we hypothesize that dietary differences did not drive the formation of stable *Prevotella*- and *Bacteroides*-dominant profiles observed in our colony. The *Prevotella*- and *Bacteroides*-dominant profiles were associated with the original source of importation, as this study included only animals that were co-housed with animals from the same original source. Interestingly, we observed that distinct gut microbiome profiles were preserved in captive marmosets fed a standardized diet through husbandry practices for a period of 2 years. In the healthy gut microbiome of captive marmosets, most bacteria observed were acetate- or propionate-producers, such as *Bacteroides*, *Prevotella, Anaerobiospirillum, Phascolarctobacterium, Megamonas,* and *Megasphaera*, with a low abundance of butyrate producers, such as *Lachnospiraceae*^48^. However, others have previously noted that *Megasphaera* can function as a butyrate producer under specific conditions^33,49^. Inter-institutional differences greatly affect marmoset microbiomes, as previous studies report marmoset gut microbiota dominated by *Actinobacteria*^29,30^, *Firmicutes*^33,34^, *Proteobacteria*^38,50,51^ and *Bacteroidetes*^31,32,51^. At the Biomedical Primate Research Centre (BPRC) (Rijswijk, the Netherlands), *Actinobacteria*, represented by *Bifidobacterium* and *Collinsella*, was the most abundant phylum (66%), while *Bacteroides* and *Prevotella* each represented < 5% of the microbiome^29^. BPRC marmosets have access to outdoor and indoor enclosures, as well as food enrichment, such as insects and gums, provided several times a week^29^. We hypothesize that increased environmental exposure and enrichment promote a wild-like microbiome, rich in bifidobacteria that help metabolize oligosaccharide-rich tree gums, a common food source for wild marmosets^50,52^. High abundances of *Actinobacteria* are observed in wild callitrichids, but not in captive and semi-captive marmosets^38^. Unexpectedly, Ross et al. also reported high *Bifidobacterium* levels in marmosets housed within a specific-pathogen free (SPF) barrier facility at the Southwest National Primate Resource Center (SNPRC)^30^. In contrast to the BPRC, these SPF marmosets fed exclusively irradiated commercial feed (Harlan Teklad), nuts, seeds, and dried fruits had median *Bifidobacterium* abundances of 17%^30^. This abundance was much higher than the non-SPF parent colony at SNPRC, which had median *Bifidobacterium* frequencies of 4% and high levels of *Fusobacterium*^30^*.* However, a follow-up report from the SPF barrier facility showed bacterial shifts with an increased *Bacteroidetes* abundance (35%) and a slight decrease in *Bifidobacteriaceae* (12%)^31^. In another colony with a microbiome similar to the MIT marmoset profile, microbiome synchronization was observed within a year in marmosets imported from another captive marmoset colony, characterized by expansion of *Bacteroidetes*, but in contrast to our study, the imported marmosets were co-housed with the original colony^51^. Imported cohorts retained unique features following microbiome synchronization^51^, supporting our findings that source-specific microbiomes can persist despite standardization of husbandry and diet. These studies demonstrate that clinically healthy captive marmosets can have multiple, stable microbiome profiles that are influenced by each institution’s housing, diet and husbandry practices. While the results of previous studies can support the hypothesis that captivity alters the marmoset microbiome into diverse microbiome profiles, further studies need to evaluate whether these differences could be accounted for by the lack of standardization in 16S rRNA primers, library preparation, and bioinformatic pipelines as the analysis of the microbiome may be affected by differences in methodology.

In spite of the reported diversity in marmoset microbiome across institutions, IBD is reported frequently in captive marmoset colonies worldwide. While research in humans implies a potential role for the microbiome in IBD, further research is necessary to determine whether a single or multiple dysbiotic states cause marmoset IBD. In this study, IBD was prevalent in marmosets from all sources, with an increased prevalence in MIT^CL^ marmosets. However, a single dysbiotic microbial signature for IBD was not evident in our analysis. Across the sources evaluated, the microbiome of marmosets with IBD exhibited similar changes. Consistent with human studies, marmoset IBD decreased alpha diversity^21,25,40^. Within each source population, IBD progressors had higher average abundances of *P. copri* and *Megamonas,* as well as decreased abundance of *Bacteroides*, relative to healthy marmosets from the same source*.* Our RF models also highlighted *Sutterella*, bacteria associated with negative fecal microbiota transplantation outcomes, shorter remission periods in UC patients^53,54^, and its ability to dampen immune responses^55^. *Megamonas,* along with *B. plebeius*, deregulate BA metabolism in CD patients^56^, which could cause dysbiosis and opportunistic pathogen infections. However, while *Megamonas* increases were observed*, Bacteroides* decreased in marmoset IBD. Most *Bacteroides* reads matched *B. plebeius*, a non-*B. fragilis* group species^57^. *B. plebeius* Amplicon Sequence Variants (ASVs) were the most abundant in the two *Bacteroides*-dominated cohorts, and only 20% of *Bacteroides* reads matched members of the *B. fragilis* group, the most frequently isolated and virulent species in clinical specimens^58^. Furthermore, the role of the *B. fragilis* group in IBD is inconclusive, as they both modulate immunity and cause infections^21,58–60^.

While the effects of *Bacteroides* and *Prevotella* spp. in IBD patients is not understood^21,61,62^, *Prevotella* have been considered inflammophilic pathobionts, commensal bacteria known to thrive in inflammatory environments and promote inflammatory diseases, such as periodontitis, bacterial vaginosis, rheumatoid arthritis (RA), and metabolic disorders^63–65^. *Prevotella*, including *P. copri*, activate TLR2, elicit specific IgA and IgG responses and promote the release of IL-1, IL-8, IL-6, IL-17, IL-23, and CCL20, which leads to neutrophil recruitment, reduced T helper 2 (Th2) cells and induction of Th17 cells^63–67^. In the gut, *Prevotella* has been linked to diarrhea, HIV-induced gut dysbiosis, irritable bowel syndrome and more severe colitis^68–70^. In a small study, higher levels of *Prevotella* were observed in marmosets with IBD compared to controls^51^. Furthermore, models of RA and colitis have shown that transfer of *Prevotella*- or *P. copri-*rich microbiota to mice transmitted disease phenotypes^64,67,68^. A possible mechanism could be linked to cycles of expansion and relaxation observed in *P. copri* abundance in healthy individuals, but absent in IBD patients^23^. Constant *P. copri* signals might promote chronic inflammation, but natural control of *P. copri* in the microbiome might prevent disease-causing chronic inflammatory states. In our study, IBD-associated enteritis upregulated pro-inflammatory immune responses in the duodenum and jejunum. Multiple genes associated with NK cell functions were upregulated by IBD, including genes associated with high cytolytic effector activity, cytotoxicity and IFN-γ production (*CD244*, *CD160, IL18R1*, *FYN*, and *IFNG*)^71,72^. In addition to *IFNG*, genes associated with Th1 cells (*TBX21*, *CCR2, CCR5,* and *IL2RB*) were also upregulated. In humans, killer immunoglobulin receptor (KIR) polymorphisms have linked NK cells with CD^73^. Further studies are needed to determine if *P. copri* causes enteritis and IBD in marmosets via NK cells.

The resilience of the gut flora to perturbations caused by captivity and its stresses in marmosets is unknown. In other NHP, wild-like microbiota may prevent captivity-associated illnesses^36^. In this study, we evaluated a marmoset colony with a human-like or “humanized” microbiota^36^ and compared the microbiota of clinically healthy individuals with marmosets with IBD. As observed in humans, a range of stable microbiome profiles may exist in clinically healthy marmosets. In this study, we determined that source-specific microbiomes can be observed in marmosets fed the same diet and housed in the same facility, but we did not evaluate the effect of co-housing individuals from different sources. Due to the natural occurrence of IBD in captive marmosets^6^, we compared the microbiome of marmosets diagnosed with IBD with our healthy cohorts and observed a decrease in alpha diversity and a lower ratio of *Bacteroides:P. copri* in diseased marmosets. Our RF model of blood parameters also supports the validity of the marmoset IBD model, as it highlighted the importance of calcium, hemoglobin and RBCs, which align with the common diagnosis of anemia and calcium deficiency in human IBD patients^74^. As observed in our captive marmoset colony, the marmoset microbiome is “humanized” and resembles the human microbiome^15,36^. The prevalence of Bacteroidetes in our colony contrasts the gut microbiome reported in wild common marmosets^38^. In addition to being an animal model for naturally occurring *P. copri*-mediated IBD in a non-human primate, the “humanized” microbiome may provide important insights into the role of the microbiome in other areas of active research involving the marmoset model, such as neuroscience, aging, and toxicology.

## Materials and methods

### Ethics statement

All research was conducted under an animal use protocol approved by the MIT Institutional Care and Use Committee (IACUC). The facility where this research was conducted is accredited by the AAALAC, International and adheres to principles stated in the Guide for the Care and Use of Laboratory Animals. Methods were carried out in accordance with the ARRIVE guidelines. Animals are cared for by a large staff of highly qualified veterinarians, veterinary technicians, and animal caretakers, who undergo substantial training to ensure only the highest quality animal care and use.

### Animals

Common marmosets (*Callithrix jacchus*) were housed at the Massachusetts Institute of Technology in Cambridge, MA, from marmosets sourced from the New England Primate Research Center (NEPRC), an international primate center (CLEA Japan Inc.), and two companies (A and B). Subsequently, the four sources will be referred to as MIT^NE^, MIT^CL^, MIT^A^, and MIT^B^. All animals were housed in pairs or family groups within two vivaria at MIT, an AAALAC International accredited program. All marmosets included in this study were on an animal use protocol approved by the MIT Institutional Care and Use Committee (IACUC).

The animal holding room temperature was maintained at 74.0 ± 2°F with a relative humidity of 30–70%. The light cycle was maintained at a 12:12 h light:dark cycle. Marmosets were housed in cages composed of stainless-steel bars and polycarbonate perches with the following dimensions: 30″ W × 32″ D × 67″ H). Each cage had a nest box made of polycarbonate attached the outside of the cage. Other cage furniture present in the cages included hammocks, hanging toys, and manzanita wood branches. Foraging enrichment in the form of dried acacia gum-filled branches and forage board were provided weekly. Cages were spot-cleaned daily and removed for sanitization on a biweekly rotation.

All animals received a base chow diet of biscuits (Teklad New World Primate Diet 8794). Biscuits were soaked in water for either a minimum of 20 min or briefly using a pour-on/pour-off soak only. In addition to the base chow, a cafeteria-style supplemental offering of fruits (e.g. bananas, blueberries, mangoes, apples and grapes), vegetables (e.g. carrots, vegetable blend), acacia gum, and additional protein sources including hard-boiled eggs, mealworms, cottage cheese or ZuPreem (Premium Nutritional Products, Inc., Mission, KS).

On a semiannual basis, preventative health physical exams were performed on all colony animals. Rectal swabs and fecal samples were collected and screened for potentially pathogenic bacteria (including *Salmonella* spp., *Shigella* spp, beta-hemolytic *E.coli*, *Klebsiella* spp., and *Campylobacter* spp.)^75^ and parasites (including *Enterobius* spp., *Entamoeba* spp., *Giardia* spp., *Taenia* spp., and *Cryptosporidium* spp.). Intradermal testing for *Mycobacterium tuberculosis* was performed semiannually as well. All animals derived from progenitor stock were negative for squirrel monkey cytomegalovirus, *Saimiriine herpesvirus 1, Saimiriine* *herpesvirus 2, *and measles virus. Complete blood count and serum chemistry analysis were performed on an annual basis and during diagnostic workup of clinical cases. Hematology analysis was performed by the MIT DCM diagnostic laboratory using a HemaVet 950 veterinary hematology analyzer (Drew Scientific, Oxford, CT). Serum chemistry analysis was performed by Idexx Laboratories (Westbrook, ME). Serum chemistry and complete blood counts data were collected from the clinical records from the MIT colony. Fecal (n = 223) and rectal swab (n = 342) were collected from common marmosets (*Callithrix jacchus*) (n = 565 samples, 173 individuals) between 2016–2018, and ages ranged from 0.19 to 11.73 years old for healthy marmosets and 0.56–13.49 years old for IBD marmosets. Of the animals evaluated in this survey, 85 were male and 88 were female. Based on medical records, history of vomiting or diarrhea, serum chemistry, weight, complete blood counts, and regular usage of budesonide or sucralfate, the 173 marmosets were categorized as healthy (n = 91), IBD (n = 59) and other disease (n = 23). For this study, only samples from healthy and IBD animals were processed for further microbiome analysis in this study. Investigators collecting samples were aware of health status, but investigators processing samples were blinded.

### 16S microbiome profiling

Fecal DNA was extracted using the DNeasy PowerLyzer PowerSoil Kit, and DNA was amplified using universal primers of F515 (GTGYCAGCMGCCGCGGTAA) and R926 (CCGYCAATTYMTTTRAGTTT) to target the V4 and V5 regions of bacterial 16S rRNA fused to Illumina adaptors and barcode sequences as described previously^76^. Individual samples were barcoded and pooled to construct the sequencing library, followed by sequencing with an Illumina MiSeq instrument to generate pair-ended 300 × 300 reads. Sequencing quality was inspected using FastQC^77^. Reads were processed using QIIME 2–2018.6 within the MicrobiomeHelper v. 2.3.0 virtual box^76,78^. Briefly, primer sequences were trimmed using the cutadapt plugin^79^. Forward and reverse reads were truncated at 243 and 195 bases, respectively, prior to stitching and denoising reads into amplicon sequence variants (ASV) using DADA2. Samples with fewer than 7500 reads were excluded. ASVs present in fewer than 3 samples and with less than 24 counts were also excluded. A total of 1085 ASVs were retained after filtering. Taxonomic classification was assigned using the custom 16S V4/V5 region classifier based on the SILVA 132 database (SSU Ref NR 99)^80^. Phylogenetic trees, composition, alpha rarefaction, beta diversity metrics and ANCOM (Analysis of Composition of Microbiome)^81^ were evaluated using built-in QIIME2 functions^82^. Microsoft Excel and R (v 3.6.3 at http://www.R-project.org/) were used to perform statistical analyses and graphically represent data. Additionally R libraries phyloseq^83^, ggplot2 (2.2.1)^84^, caret^85^, vegan^86^, pROC^87^, and gtools^88^ were used to model microbiome data. Samples that were determined to exhibit deficient sampling were excluded from microbiome analysis based on criteria previously outlined, such as low quantities of visible fecal matter on a swab, a microbiome dominated by a single species (e.g. *Helicobacter*), and discordance from paired samples collected from the same individual^32^. The *Bacteroides*/*Prevotella* abundance ratio was calculated using the ratio of the averaged *Bacteroides* abundance and the averaged *Prevotella* abundance.

### Machine learning

Data from the microbiome, serum chemistries and complete blood counts were utilized to train classifiers. Data was normalized using min–max normalization. The data was then split using a single partition method and the classifiers were trained on 80% of the samples (training). The training set was associated with the sample’s classification (source or health status), and the discovered signatures were used to predict the populations on the remaining 20% of samples (testing) using the four machine learning approaches: support vector machines (SVM), random forest (RF), K-nearest neighbor (KNN), and Classification and Regression Trees (CART). Comparison of each model’s predictions on the testing data against actual sample classifications were used to determine the model’s accuracy. The model generation process was carried out iteratively to sample multiple training/testing subsets of the data and determine the robustness of the algorithm. A R script using the function in the Caret package utilized default parameters for training with cross-validation. The variable importance metric was calculated using the varImp function, which associated a specific value for each parameter. To evaluate the contribution of each parameter, the script ranked the parameters and calculated the variable importance starting with the ranked parameters with the highest score. This process was processed iteratively adding ranked parameters and recalculating the metrics with each subsequent addition until all ranked genes were evaluated. Metrics included accuracy (correct classification percentage compared to ground truth data), kappa value (inter-rater classification agreement), sensitivity, specificity, precision, recall, prevalence, and F1 score (harmonic average of the precision and recall). Based on the contribution of each parameter, we selected a K value of top parameters based on the following criteria: (i) the stability of the metrics (priority for accuracy, kappa, and F1) when the increment of ranked genes was done, and (ii) minimum number K of parameters as possible. After the selection of the K value, ROC (Receiver-operating characteristic) curve and AUC (Area under the curve) value were calculated for each algorithm.

### RNAseq

Tissues were collected from the jejunum from marmosets during necropsies performed by clinical veterinarians and veterinary pathologists. Based on pathological analysis and clinical presentation, marmosets were classified as IBD or non-IBD. In IBD cases, the jejunum presented with increased thickening (n = 3), while in non-IBD cases, the jejunum was grossly normal (n = 3). These observations were confirmed by histopathological analysis. Tissues were flash frozed in liquid nitrogen and stored at − 80 °C. RNA was extracted using TRIzol reagent according to manufacturer’s instructions (Thermo Fisher Scientific). Total RNA was shipped on dry ice to Arraystar, Inc. (Rockville, MD) for quality control, rRNA depletion and sequencing on an Illumina HiSeq4000. FASTA files and the NCBI RefSeq GTF files for *Callithrix jacchus* based on the March 2009 (WUBSC 3.2/calJac3) assembly were obtained from the UCSC Genome browser^89^. Raw sequencing reads were mapped to an index built from *C. jacchus* FASTA files using Rsubread^90^. Feature counts were obtained from the bam files using annotated exons in the *C. jacchus* GTF files. Analysis was then performed using edgeR^91,92^. Lowly expressed exons were removed using a cutoff of 10 counts per million (CPM). Normalization was performed using the Trimmed Mean of M-values (TMM) method. Multidimensional scaling (MDS) plots and heatmaps were used to evaluate grouping of biological samples. Data was fitted using the glmQLFit function that uses a generalized linear model (GLM) implementing a quasi-likelihood (QL) fitting method. Quasi-likelihood F-tests were performed to test for differential expression based on False Discovery Rate (FDR) adjusted P-values of 0.05. To retrieve Gene Ontology (GO) classifications, *C. jacchus* genes that matched *Homo sapiens* gene names were assigned both the *C. jacchus* and *Homo sapiens* Entrez IDs. GO analysis was performed using limma^93^, AnnotationDbi^94^, GO.db^95^, topGO^96^, mygene^97^ and org.Hs.eg.db. Data was visualized using ggplot2, gplots, Rgraphviz^98^, colorspace^99^ and ggVennDiagram^100^. Analysis of the IBD dataset demonstrated that the expression profile of one sample differed from the remaining samples and was excluded from the analysis presented.

## Supplementary Information

Supplementary Figure 1.

Supplementary Figure 2.

Supplementary Figure 3.

Supplementary Figure 4.

Supplementary Figure 5.

Supplementary Figure 6.

Supplementary Figure 7.

Supplementary Tables.

## Data Availability

RNAseq data is available under NCBI GEO accession number GSE156839. Microbiome data is available under NCBI BioProject PRJNA659472.

## Code availability

R scripts utilized to analyze data and generate figures are available at https://github.com/sheh-dcm/cj_microbiome.
