# Supplementary figures and images for: Analysis of gut microbiome profiles in common marmosets (Callithrix jacchus) in health and intestinal disease
Source: Sci Rep. 2022 Mar 15;12:4430. doi: 10.1038/s41598-022-08255-4 (PMC8924212; doi:10.1038/s41598-022-08255-4)

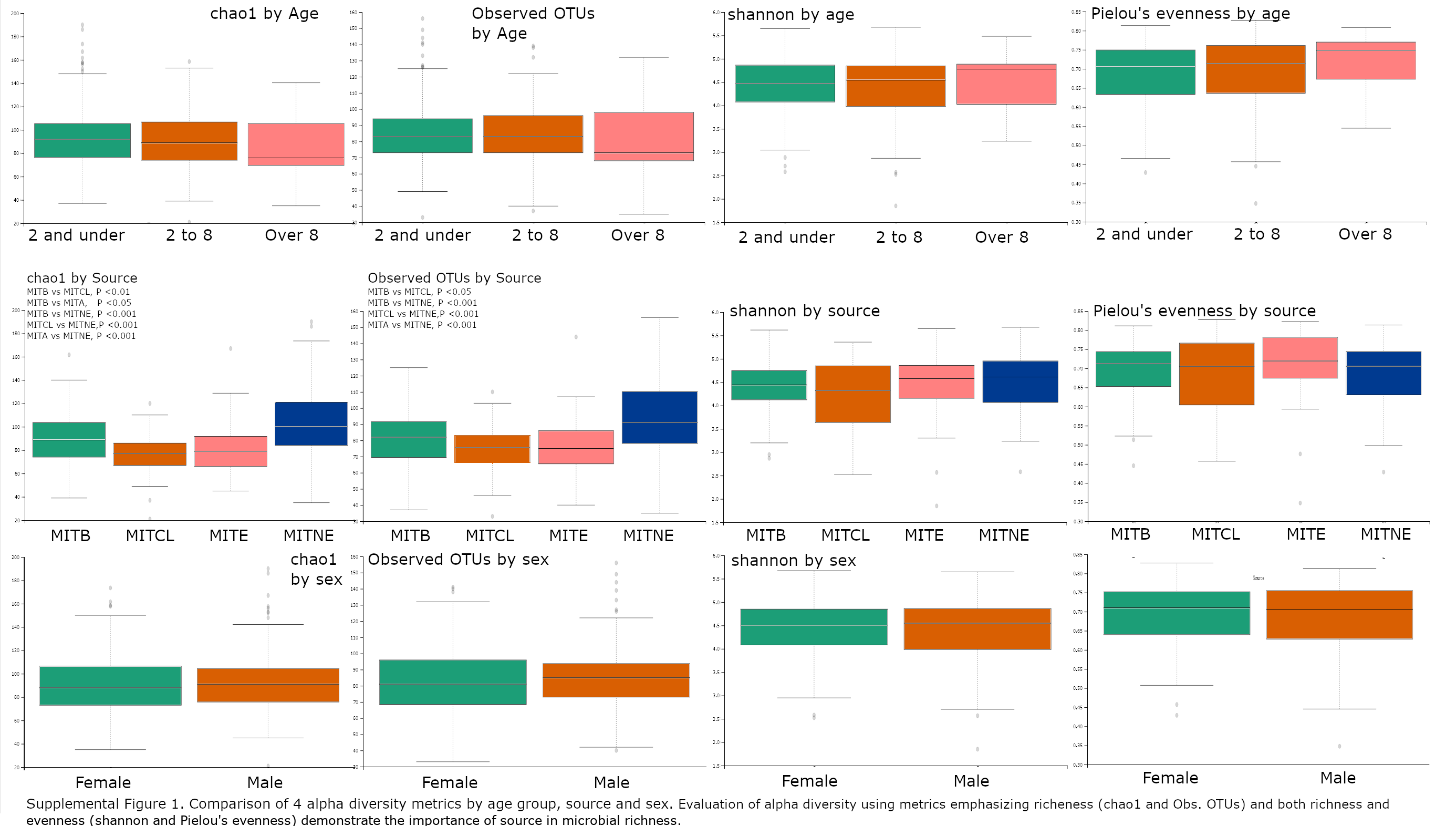

Supplement: Supplementary file 1 — Supplementary Figure 1. [file 41598_2022_8255_MOESM1_ESM.tiff]

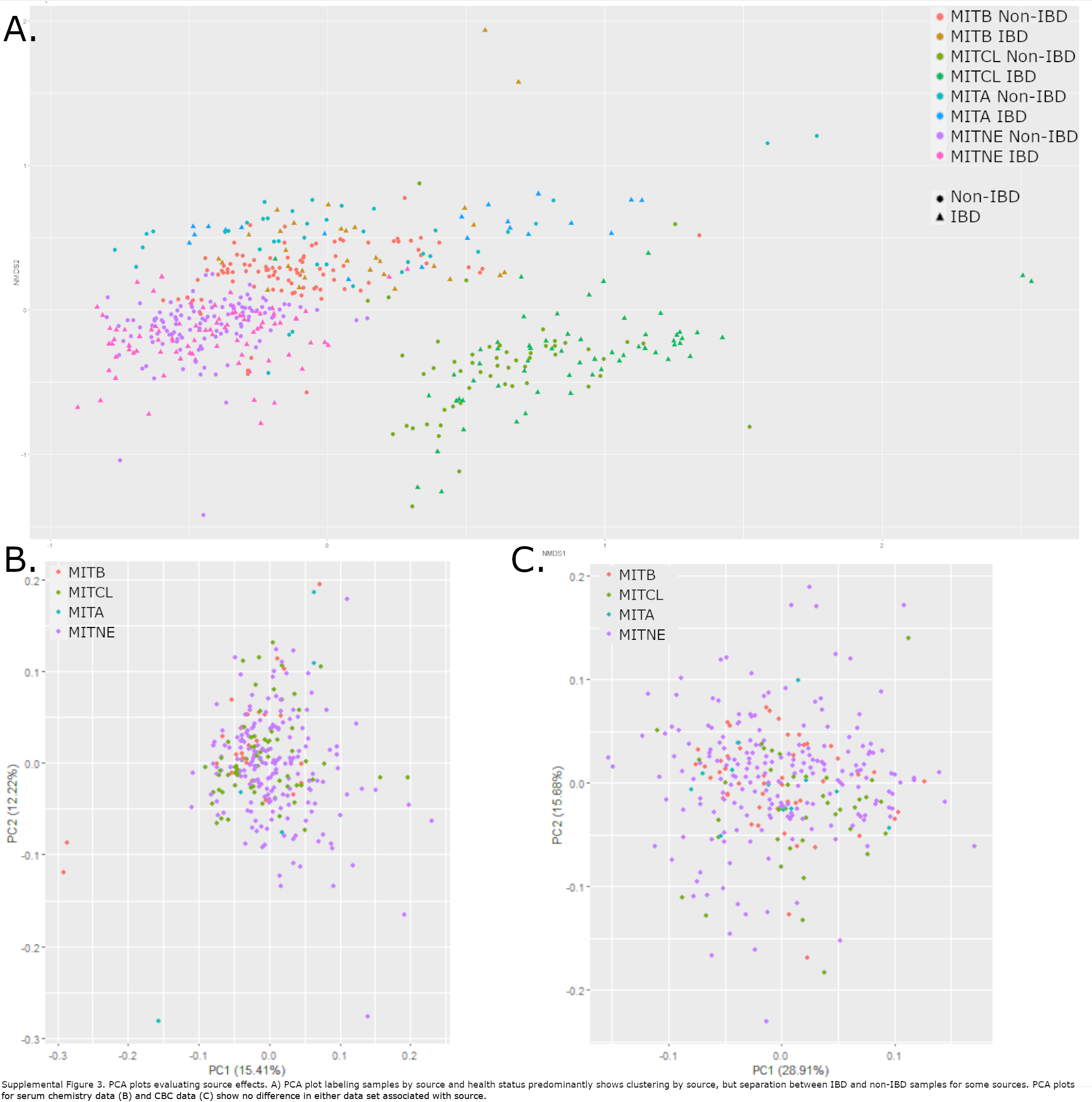

Supplement: Supplementary file 3 — Supplementary Figure 3. [file 41598_2022_8255_MOESM3_ESM.tiff]

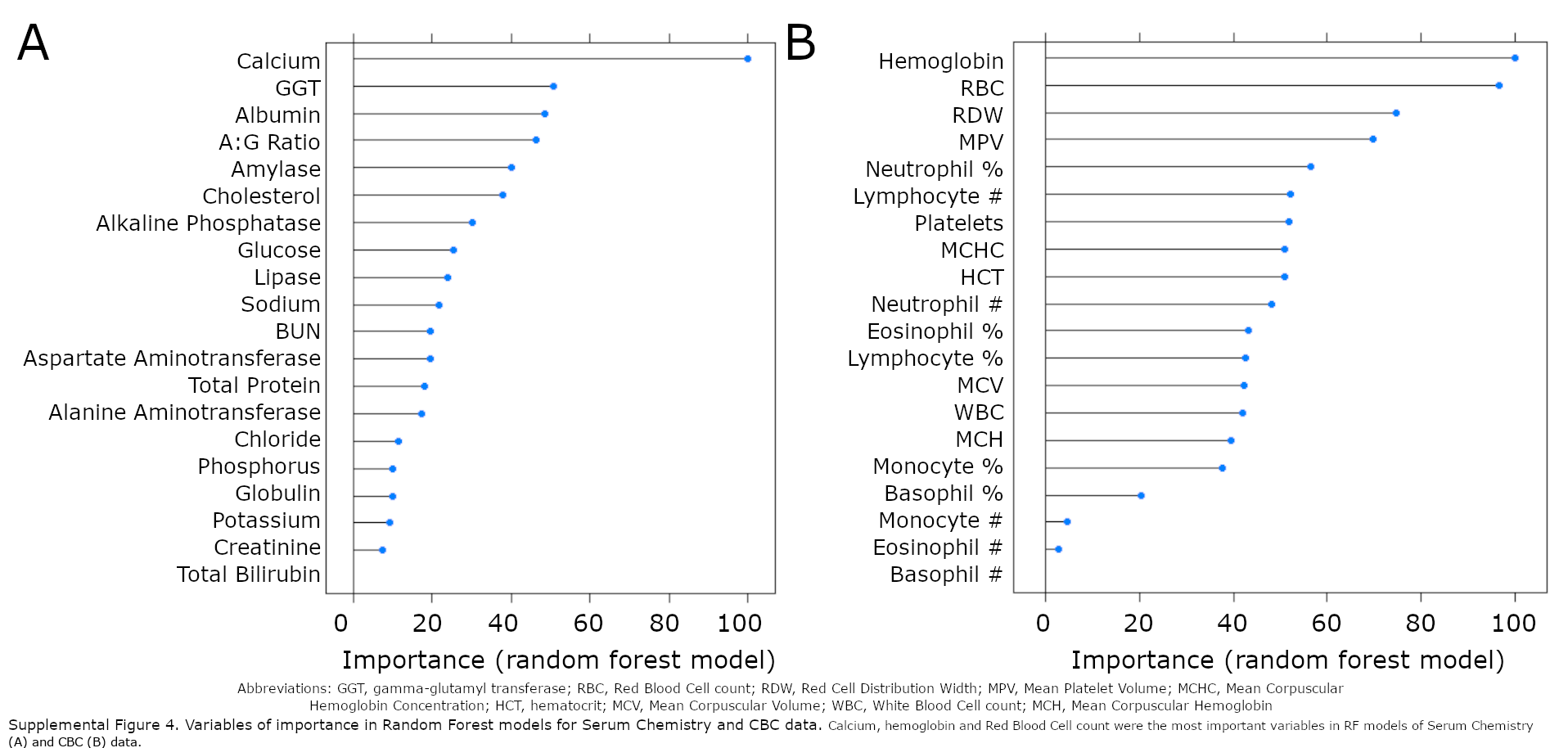

Supplement: Supplementary file 4 — Supplementary Figure 4. [file 41598_2022_8255_MOESM4_ESM.tiff]

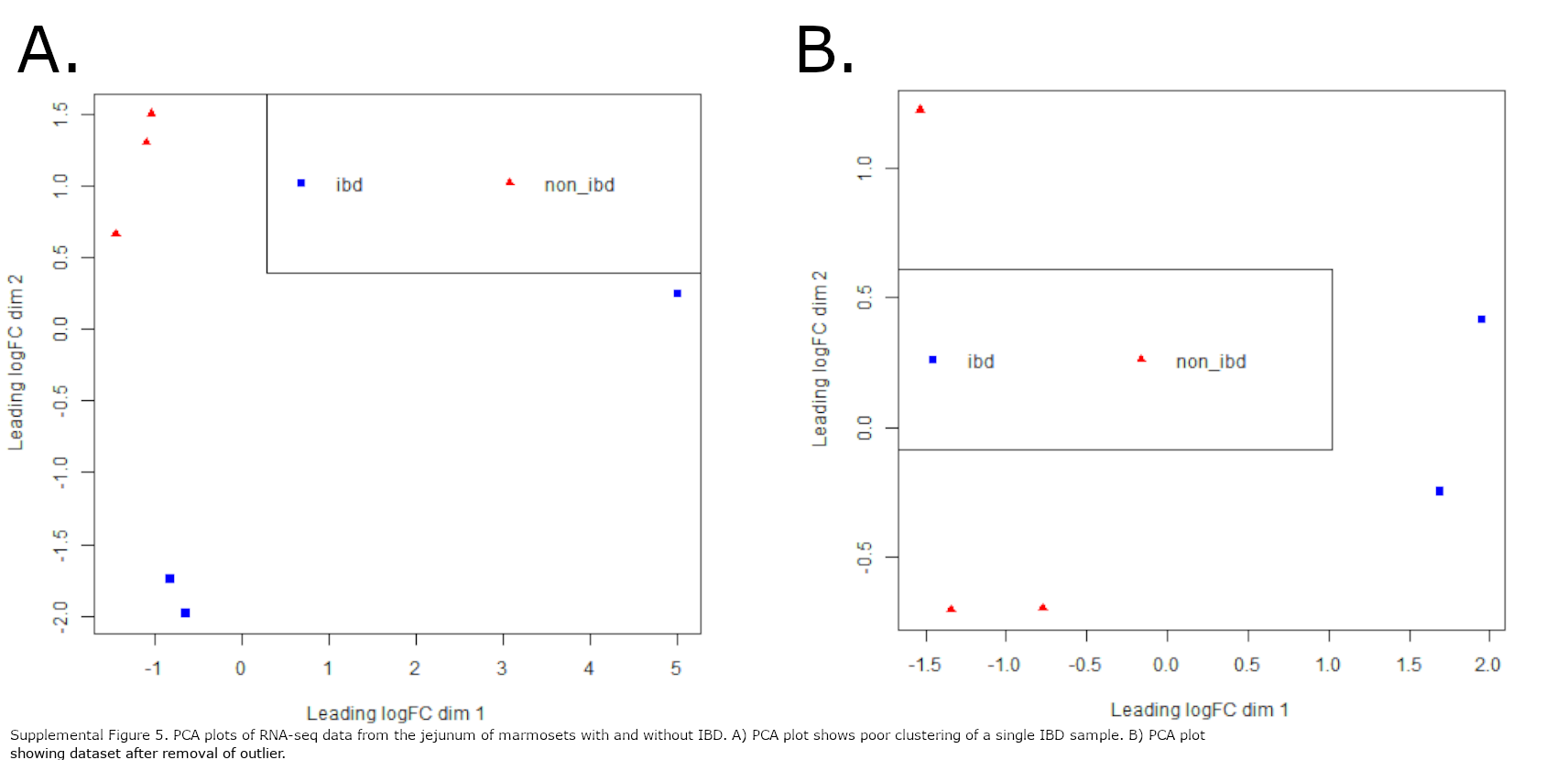

Supplement: Supplementary file 5 — Supplementary Figure 5. [file 41598_2022_8255_MOESM5_ESM.tiff]

Supp. Fig 6 Biological Processes enriched in the jejunum of IBD cases

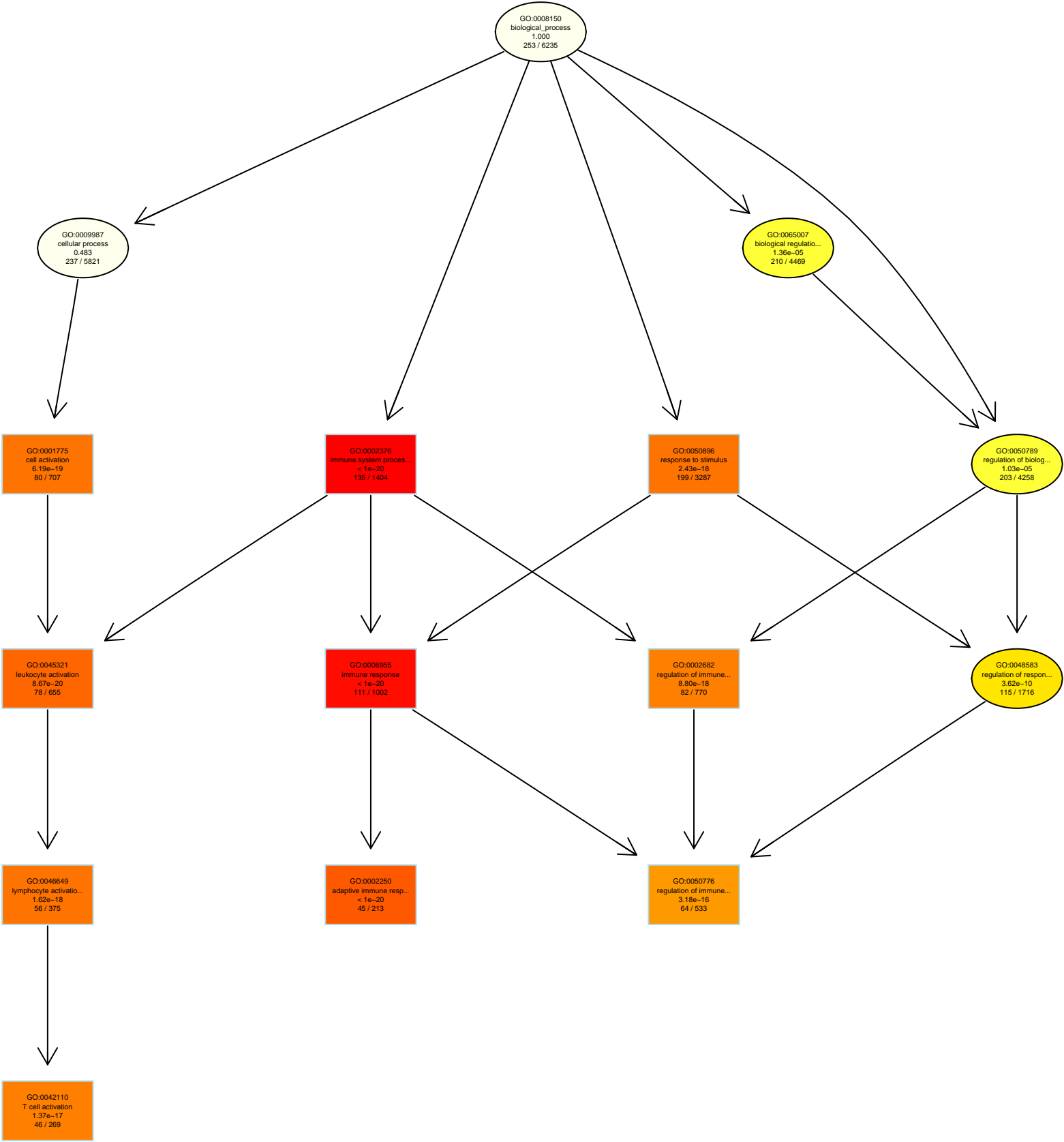

Supplement: Supplementary file 6 — Supplementary Figure 6. [file 41598_2022_8255_MOESM6_ESM.pdf]

Supp. Fig. 7 Biological processes enriched  
in the jejunum of non-IBD cases

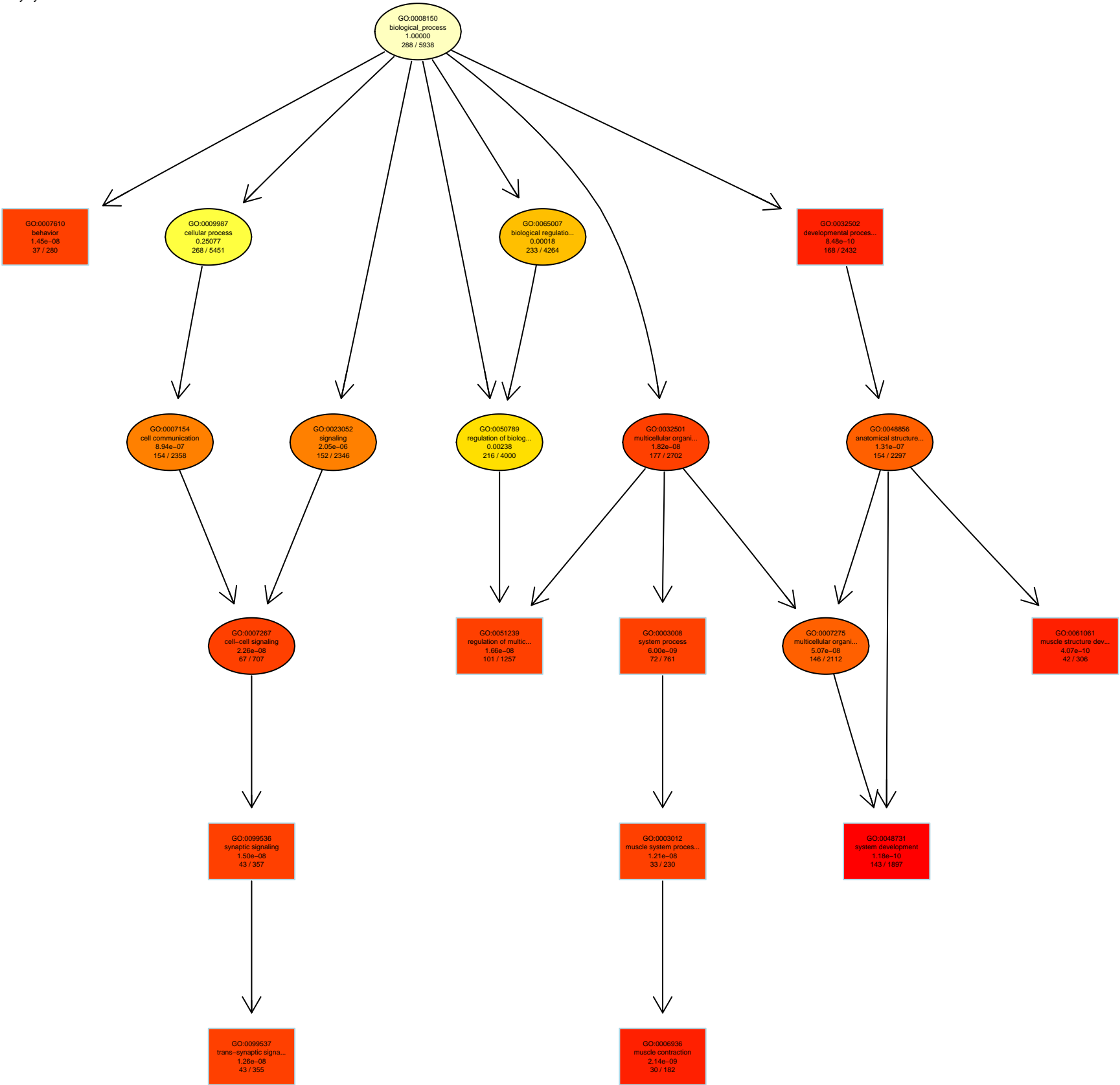

Supplement: Supplementary file 7 — Supplementary Figure 7. [file 41598_2022_8255_MOESM7_ESM.pdf]
